# Capsular Polysaccharide is a Main Component of *Mycoplasma ovipneumoniae* in the Pathogen-Induced Toll-Like Receptor-Mediated Inflammatory Responses in Sheep Airway Epithelial Cells

**DOI:** 10.1155/2017/9891673

**Published:** 2017-05-03

**Authors:** Zhongjia Jiang, Fuyang Song, Yanan Li, Di Xue, Guangcun Deng, Min Li, Xiaoming Liu, Yujiong Wang

**Affiliations:** ^1^Key Laboratory of Ministry of Education for Conservation and Utilization of Special Biological Resources in Western China, Ningxia University, Ningxia 750021, China; ^2^College of Life Science, Ningxia University, Yinchuan, Ningxia 750021, China; ^3^Institute for Stem Cell Research, General Hospital of Ningxia Medical University, Yinchuan, Ningxia 750004, China

## Abstract

*Mycoplasma ovipneumoniae* (*M. ovipneumoniae*) is characterized as an etiological agent of primary atypical pneumonia that specifically infects sheep and goat. In an attempt to better understand the pathogen-host interaction between the invading *M. ovipneumoniae* and airway epithelial cells, we investigated the host inflammatory responses against capsular polysaccharide (designated as CPS) of *M. ovipneumoniae* using sheep bronchial epithelial cells cultured in an air-liquid interface (ALI) model. Results showed that CPS derived from *M. ovipneumoniae* could activate toll-like receptor- (TLR-) mediated inflammatory responses, along with an elevated expression of nuclear factor kappa B (NF-*κ*B), activator protein-1 (AP-1), and interferon regulatory factor 3 (IRF3) as well as various inflammatory-associated mediators, representatively including proinflammatory cytokines, such as IL1*β*, TNF*α*, and IL8, and anti-inflammatory cytokines such as IL10 and TGF*β* of TLR signaling cascade. Mechanistically, the CPS-induced inflammation was TLR initiated and was mediated by activations of both MyD88-dependent and MyD88-independent signaling pathways. Of importance, a blockage of CPS with specific antibody led a significant reduction of *M. ovipneumoniae*-induced inflammatory responses in sheep bronchial epithelial cells. These results suggested that CPS is a key virulent component of *M. ovipneumoniae*, which may play a crucial role in the inflammatory response induced by *M. ovipneumoniae* infections.

## 1. Introduction

Mycoplasma bacteria have been extensively studied as one class of the smallest, free-living, and self-replicating bacterial cells [1]. Pathogenically, they have an ability to colonize the respiratory epithelium by attaching to the cilia of epithelial cells therefore resulting in clumping and loss of cilia and the subsequent decrease in mucociliary apparatus function [2–5]. Although attachment is of critical in the pathogenic process by causing a physical damage, it is insufficient to explain all aspects during mycoplasma infection. Numerous factors appear to act in synergy for the occurrence and development of disease during an infection of mycoplasma bacteria. For instance, immunopathological lesions associated with *Mycoplasma pneumoniae* are characterized by an infiltration of the perivascular and peribronchiolar spaces with lymphocytes and macrophages, suggesting an involvement of immunomodulatory effects in the host respiratory immune system [6–9].

*Mycoplasma ovipneumoniae* (*M. ovipneumoniae*) is an etiological pathogen that colonizes respiratory epithelial cells of sheep exposed to contaminated air and is known to be a major contributing factor to the occurrence of primary atypical pneumonia [2, 3, 10–13]. Polysaccharides, polymers of repeated mono- or di-oligosaccharide residues joined together by glycosidic bonds, are main component of the outer capsule of *M. ovipneumoniae* which play significant roles in its growth and development [14–16]. Capsular polysaccharide (designated as CPS) is cell-surface polysaccharide that is one of the few identified virulent determinants and considered to mediate interactions with host cells. It has been shown to possess various biological activities such as functions in cellular adherence, invasion, virulence, and immune modulation [17]. These functions are considerably contributing to the pathogenic mechanism of *M. ovipneumoniae*. Recent studies have shown that CPS of *Mycoplasma pneumoniae* could be recognized and subsequently combined with specific receptors on dendritic cells and macrophages, with the purpose of enhancing their viabilities against pathogenic microorganisms by promoting the secretion and expression of proinflammatory mediators, such as interleukin 10 (IL10) and IL12 [18]. However, limited information concerning its immunomodulating activities and molecular pathogenesis is currently available during *M. ovipneumoniae* infection. As a consequence, it is of meaning to explore the proinflammatory effects and precise molecular mechanisms of the capsular polysaccharides from *M. ovipneumoniae.*

Airway epithelium has recently been recognized as the first line of defense against *M. pneumoniae* infection, which is responsible for initiating an innate immune response by producing various array inflammatory mediators thus mediating lung inflammation [15, 19, 20]. Additionally, it plays a key role in regulating adaptive immune responses via expressing pattern recognition receptors such as toll-like receptors (TLRs) to trigger host defense responses, by interacting with dendritic cells to regulate antigen sensitization and by releasing cytokines and chemokines to recruit effector cells [19, 21]. Therefore, airway epithelial cells serve as a bridge between innate and adaptive immunity via acting as initiators, mediators, and regulators. These evidences of roles of airway epithelium cells as immune mediators and effectors clearly indicate their involvement in inflammatory responses upon *M. ovipneumoniae* infection.

However, most of previous colonization models of *M. ovipneumoniae* infection employed with conventional monolayer culture of epithelial cells, rather than the multilayered and differentiated pseudostratified cells with polarized cilia and mucus secretion, are limited in their ability to accurately display the functions of ciliated cells [22–24]; therefore, they might not fully reflect the nature immune responses of respiratory epithelial cells in response to the mycoplasma infection. One the other hand, the air-liquid interface (ALI) culture models generated with primary airway epithelial cells of various species including human [25], bovine [26], and sheep [27] have been developed and used in studies of physiology and biological functions, which allows an improved mimicking of pathogen-host interactions occurring in vitro. Therefore, in the present study, the molecular mechanism of proinflammatory responses in bronchial epithelial cells of Tan sheep to *M. ovipneumoniae*-derived CPS was investigated using an ALI model.

## 2. Materials and Methods

### 2.1. Ethic Statement

The present study was approved by the ethics committee for the care and use of animals at Ningxia University.

### 2.2. Propagation of *M. Ovipneumoniae* and Purification of CPS

The *M. ovipneumoniae* Queensland Strain Y98 was cultured and propagated in a mycoplasma broth base CM403 culture media, supplemented with supplement-G SR59 (OXOID, Hampshire, UK), and 0.002% phenol red as well as an additional 10% glucose so as to maximize the production of polysaccharide. The preparation of *M. ovipneumoniae* CPS was carried out according to the method of Allen et al. with slight modification [28]. Briefly, the cell pellets were treated with phenol water at 60°C by a constant stirring for 30 min to remove lipophilic substances and other compounds with low molecular weight and then centrifuged at 5000 × g for 30 min at room temperature. The upper aqueous phase was then collected and dialyzed using a cellulose ester tubular membrane (exclusion limit 3500 Da), followed by treatment with DNase I, RNase, and pronase K. Afterwards, the resulting solution was sequentially subjected to extract with phenol/chloroform (1 : 1) and chloroform using a Phase-lock gel (Tiangen, Beijing, China) in order to remove residual lipophilic components of cytoplasmic membranes. The final crude polysaccharide extract (designated as CPS) was obtained by precipitation with 4.0-fold volume of cold ethanol containing 3 M sodium acetate at 4°C overnight. Above crude CPS was dissolved in distilled water and fractionated for purification using a DEAE-cellulose anion-exchange chromatography column (1.0 cm × 5.0 cm; GE Healthcare Bio-Science AB, Uppsala, Sweden) equilibrated with distilled water. Polysaccharides were eluted with distilled water and NaCl gradient (from 0-1.0 M) at a flow rate of 0.5 mL/min. The eluent was then collected using an automatic collector (300 *μ*L/tube) and detected by an improved phenol-sulfuric acid method at 490 nm. The elution peaks were finally evaluated, and the main fraction was collected as purified CPS, which was stored at −80°C after lyophilization. As the preferable effect of CPS on cell viability was optimized in our previous studies, 100 ng/mL of CPS for 48 h was used in the following experiments.

### 2.3. Production of Polyclonal Antibodies to CPS of *M. Ovipneumoniae*

Polyclonal antiserum against purified CPS was raised in two female-specific pathogen-free New Zealand white rabbits (1.5–2.0 kg). Considering the low immunogenicity of pure polysaccharide, above purified CPS was first coupled with a carrier protein bovine serum albumin (BSA) to improve its immunogenicity. The rabbits were firstly injected subcutaneously with 1 mL of CPS-BSA-conjugated antigen solution emulsified in Freund's complete adjuvant (Sigma, St. Louis, MO, USA) and then boosted with another equivalent solution in Freund's incomplete adjuvant (Sigma, St. Louis, MO, USA) for three times with two weeks interval. Seven (7) days after the final boost, blood was droved and allowed to clot overnight and then centrifuged and the serum was subsequently decanted off and stored at −20°C. Afterwards, the polyclonal antibody against CPS was purified as IgG fraction from the resulted antiserum by affinity chromatography on a HiTrap Protein G column (GE Healthcare). The evaluation of antibody binding to CPS was eventually conducted using an indirect enzyme-linked immunosorbent assay (ELISA), as described previously [29].

### 2.4. Immunofluorescence Staining

*M. ovipneumoniae* was cultivated until the late stationary growth phase, and the sample was then pelleted by centrifugation for 30 min at 12000 × g. After washing three times for 10 min with phosphate-buffered saline (PBS, pH = 7.4), bacterial cells were fixed with 4% paraformaldehyde for 15 min followed by a permeabilization with 0.3% TritonX-100 for 10 min at room temperature. Nonspecific antibody binding was subsequently blocked using 3% normal BSA in PBS for 2 h at room temperature, after which rabbit anti-*M. ovipneumoniae* CPS IgG (self-made antibodies) (1 : 100) and rabbit nonspecific IgG (Beyotime, Shanghai, China) were applied and incubated in a humidified chamber at 4°C overnight. The binding of primary antibody was then detected by an addition of Rhodamine- (TRITC-) labeled goat-anti-rabbit IgG secondary antibody (Proteintech Group, Chicago, USA) (1 : 100). After extensively washing for 3 × 5 min, bacterial cells were mounted on slides and photographed using LEICA TCS SP2 A0BS Confocal system. Images were processed by Leica Confocal Software v.2.6.1 (Leica, Mannheim, Germany).

### 2.5. In Vitro ALI Culture of Sheep Bronchial Epithelium and Infection of *M. Ovipneumoniae*

ALI cultures of sheep bronchial epithelial cells were generated as previously described [27, 30]. Briefly, bronchial epithelial cells were obtained and seeded in polycarbonate/polyester porous collagen-precoated membranes (PCF Millicell inserts, Millipore, Bedford, MA) using Bronchial Epithelial Cell Growth Medium (BEGM) (Lonza, Basel, Switzerland) containing 5% fetal bovine serum (FBS) and cultured at 37°C for approximately 24–48 h. The BEGM was subsequently removed, and a 2% Ultroser G medium (Pall, NY, USA) was applied to the basolateral side of the chamber to establish an air-liquid interface (ALI) culture system. Followed by 4–6 weeks of cultivation, epithelial cells were well polarized and highly differentiated were capable of making significant infections. For a 2.4 cm diameter Millicell insert membrane, normally 1 × 10^7^ well-differentiated epithelial cells were determined [31]. For infection or treatment, *M. ovipneumoniae* cells or CPS were suspended and diluted in 2% Ultroser G medium and applied on the apical surface of ALI epithelial cells with indicated time periods until the cells and cultures were harvested for analysis. In addition, an equal volume of 2% Ultroser G medium was used as an untreated control. Normally, volumes of 0.5 mL and 1 mL were employed for coving the wells with diameters of 1.2 cm and 2.4 cm, respectively.

### 2.6. RNA Isolation and Quantitative Real-Time PCR (RT-PCR)

Total cellular RNA was extracted using the TRIzol Reagent (Invitrogen, Carlsbad, CA, USA) according to the manufacturer's instructions. The quality and quantity of RNA were ascertained by a NanoDrop system (Thermo Fisher Scientific, Waltham, MA, USA). First-strand complementary DNA (cDNA) was synthesized with the PrimeScript™ RT Master Mix Reagent Kit (TaKaRa, Tokyo, Japan) using 10 *μ*L of reaction mixture containing 500 ng of total RNA, 2 *μ*L of 5x PrimeScript reaction mix, and RNase-free water. The RT-PCR was performed at 37°C for 15 min and was stopped by heating to 85°C for 5 sec. The primers specific for the genes of interest were designed and synthesized in Sangon Biotech Inc. (Shanghai, China) using bioinformatic tools and are listed in Table 1. The quantitative real-time PCR (qRT-PCR) was performed using TransStart® Tip Green qPCR SuperMix (2x) (TransGen, Beijing, China) as follows: a precycling stage at 94°C for 30 sec, then 40 cycles of denaturation at 94°C for 5 sec, and an annealing step at 60°C for 30 sec. In a parallel experiment, each sample was tested three times and *β*-actin was used as an internal control. Fold changes over controls were calculated by a 2^−ΔΔCT^ method to determine mRNA expression levels [32].

### 2.7. Immunoblotting Analysis

6-well ALI inserts were infected with *M. ovipneumoniae* cells or treated with CPS as indicated conditions for 48 h. After extensively washing for 3 × 1 min, cells were lysed using the Whole Protein Extraction Kit (KeyGEN, Nanjing, China) according to the manufacturer's instructions and harvested by centrifugation at 12,000 ×g for 5 min at 4°C. Protein concentrations were determined by the BCA Assay kit (KeyGEN, Nanjing, China). 60 *μ*g of total protein of each lysate were then resolved by 8–12% SDS-PAGE and subsequently transferred to polyvinylidene fluoride (PVDF) membranes (Millipore, Bedford, MA, USA). Afterwards, membranes were blocked with 5% nonfat milk in TBST at room temperature for 2–4 h, followed by incubating with antibodies against protein of interest overnight at 4°C. Antibodies used in this study included rabbit anti-Toll-like receptor 4, rabbit anti-AP-1, rabbit anti-p-AP-1 (Cell Signaling Technology, Beverly, MA, USA), rabbit anti-MyD88, rabbit anti-IRAK1, rabbit anti-IRAK4, rabbit anti-TRAF6, rabbit anti-TAB1, rabbit anti-TRIF, rabbit anti-TRAF3, rabbit anti-IRF3, rabbit anti-IRF5, rabbit anti-*β*-actin (Proteintech Group, Chicago, USA), rabbit anti-IRAK2, rabbit anti-TBK1 (ABGENT, San Diego, USA), and rabbit anti-p-NFkB (Signalway Antibody, Maryland, USA). After washing with TBST for 3 × 10 min, membranes were striped with appropriate HRP-conjugated secondary antibodies for 1-2 h at room temperature, followed by visualizing using the enhanced Western Bright ECL reagents (Advansta, Menlo Park, CA, USA). Eventually, bands were imaged and densimetrically analyzed and semi-quantified by Image J software version 1.46 (NIH, Bethesda, MD, USA), as previously described [33].

### 2.8. ELISA for Cytokine Measurements

Commercial enzyme-linked immunosorbent assay (ELISA) kits obtained from Enzyme-Linked Biotechnology (Shanghai, China) were used for the measurement of TNF*α*, IL1*β*, IL6, IL8, and IL10 protein levels in culture supernatant. The 4-week ALI cultures were infected with *M. ovipneumoniae* (multiplicity of infection, MOI = 30) or treated with CPS (100 ng/mL) for 48 h, prior to the collection of the supernatant and measurement of inflammation-associated factor concentrations. Absorbance was eventually measured at 450 nm using a microplate reader (Thermo Fisher Scientific, Waltham, MA, USA) according to the manufacturer's protocols, and standard curves were used for ascertaining cytokine concentration.

### 2.9. Statistical Analysis

All data in this study were presented as the mean ± SD of data from at least three independent experiments. SPSS statistics 17.0 (SPSS Inc., Chicago, IL, USA) was used for the statistical analysis. Statistical differences between groups were performed using one-way analysis of variance (ANOVA) followed by post hoc Tukey's test. *P* values < 0.05 were considered statistically significant.

## 3. Results

### 3.1. Validation of CPS Antibody Using Immunofluorescence Staining

**Figure 1:**
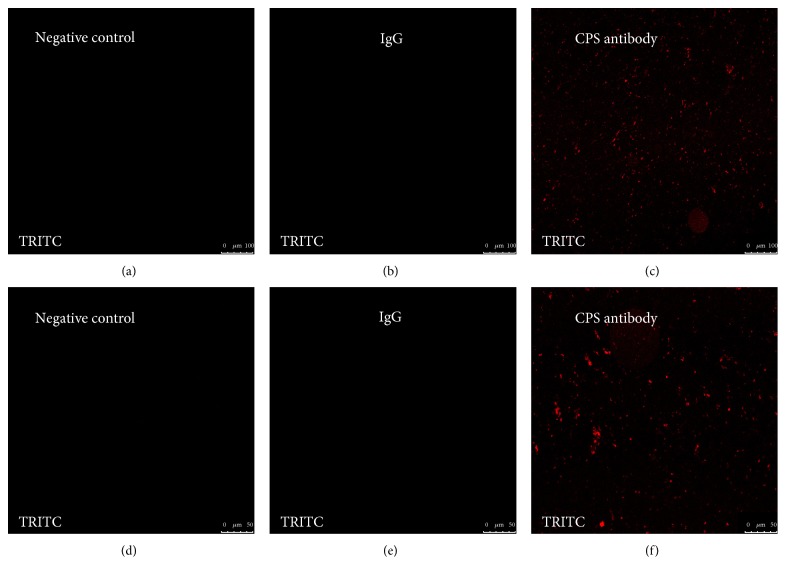
CPS antibody binds to *M. ovipneumoniae* in vitro. 1 × 10^10^ CFU (colony formation units) of *M. ovipneumoniae* solution were prepared, and the CFU value was adjusted by dilution before the test was conducted. For optimization of the immune reaction, 1 × 10^5^ CFU were investigated. The bacterial cells were incubated with rabbit anti-*M. ovipneumoniae* CPS IgG (1 : 100) and rabbit nonspecific IgG and then probed with Rhodamine- (TRITC-) labeled secondary antibody for red fluorescence. Representative images of confocal microscopy for *M. ovipneumoniae* after binding of CPS antibody indicate the presence of CPS (red) and confirm the validity of CPS antibody. Bars in (a)–(d): 100 *μ*m.

In order to assess the immunogenic potential of CPS and be able to correlate further experimental results, it is of importance to apply reliable laboratory test methods that provide valid assessments of antibody responses. To this end, immunofluoresence staining was applied in the first part of our study. The results in Figure 1 provided convincing evidence that a polyclonal antibody preparation was capable of reacting with *M. ovipneumoniae* bacterial cells, therefore showing the validity and rationality of CPS antibody. This polyclonal antibody can be further used either in immunoblotting or ELISA analysis to demonstrate its ability to recognize and neutralize CPS in cells.

### 3.2. CPS is a Key Component of *M. Ovipneumoniae*-Activated TLR Signaling in Sheep Bronchial Epithelial Cells

**Figure 2:**
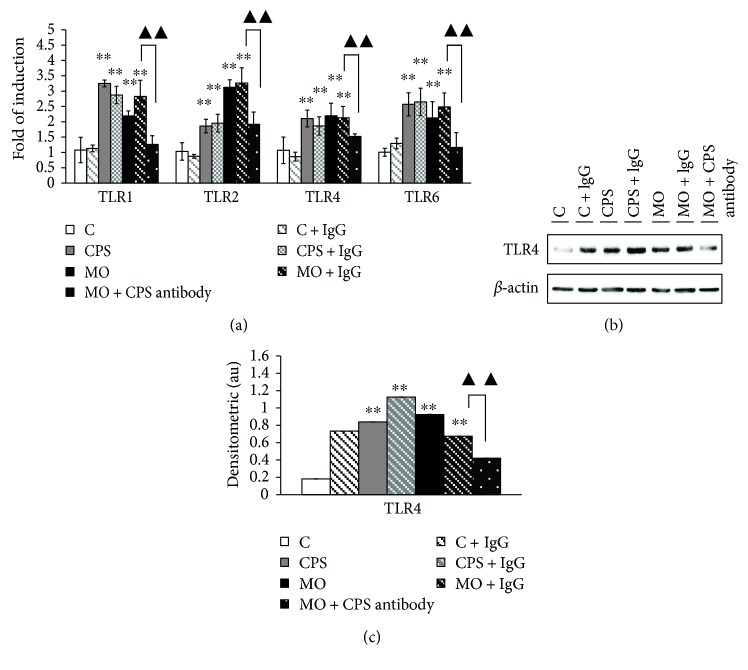
CPS stimulation induces the expression of TLRs in sheep bronchial epithelial cells*. M. ovipneumoniae* bacterial cells were preincubated with CPS antibody (1 : 100) at 4°C overnight. 4-week old ALI cultures of sheep bronchial epithelial cells were then apically treated with capsular polysaccharide (CPS) at 100 ng/mL or infected with *M. ovipneumoniae* (*MO*) at MOI of 30 for 48 h before samples were harvested for analysis. For each treatment, an additional nonspecific IgG (1 : 100) was supplemented as a control. (a) The induction of TLR1, TLR2, TLR4, and TLR6 transcripts was determined by a qRT-PCR assay. All TLR transcripts showed a significant increase in the cells stimulated with CPS or *M. ovipneumoniae*, in comparison with the controls. The expression levels of these mRNA were calculated by 2^−ΔΔCt^ method. (b) Immunoblotting assay also showed an evoked expression of TLR4 proteins in ALI cultures upon CPS/*M. ovipneumoniae* stimulation. (c) Densitometric values are shown for TLR4 proteins over *β*-actin. Values are mean ± SD for at least three independent experiments performed in triplicate. ^∗∗^*p* < 0.01 versus that of the control. Compared between indicated groups, ^▲▲^*p* < 0.01.

Considering that CPS generally plays a crucial role in the mechanism of specific recognition between host and pathogen interactions, we therefore sought to interrogate the impact of CPS treatment on TLR signaling activation in sheep bronchial epithelial cells. As expected, results showed transcripts of toll-like receptors (TLRs) including TLR1, TLR2, TLR4, and TLR6 which were significantly elevated upon CPS or *M. ovipneumoniae* stimulation (Figure 2(a)). Additionally, cells treated with CPS antibody exhibited a decreased ability to induce the expression of TLRs following *M. ovipneumoniae* infection (Figure 2(a)), suggesting an involvement of TLRs signaling in epithelial cells in response to CPS or *M. ovipneumoniae* stimulation. Consistently, the increased expression of TLR4 protein in response to an *M. ovipneumoniae* infection could be partially blocked by the addition of CPS antibody (Figures 2(b) and 2(c)).

### 3.3. The CPS Triggered a MyD88-Dependent TLR Signaling in Sheep Bronchial Epithelial Cells

**Figure 3:**
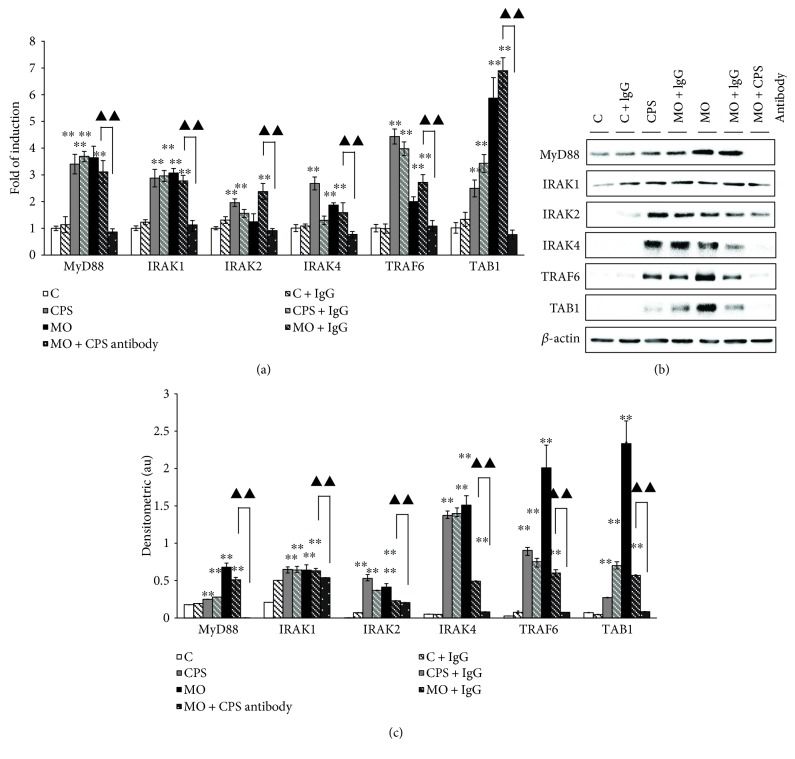
The impact of CPS on the expression of MyD88-dependent TLR signaling pathway in ALI cultures of sheep bronchial epithelial cells. 4-week old ALI cultures of sheep bronchial epithelial cells were exposed to indicated conditions for 48 h. (a) The expression of several key components of MyD88-dependent signaling cascade, including the MyD88, IRAK1, IRAK2, IRAK4, TRAF6, and TAB1 transcripts were determined by qRT-PCR assays. In comparison with the controls, all tested transcripts showed a significant increase in the cells treated with CPS or *M. ovipneumoniae*. (b) Immunoblotting assay also showed an evoked expression of all MyD88-dependent signaling-associated proteins in ALI cultures upon CPS/*MO. ovipneumoniae* stimulation. (c) Densitometric analysis of western blot showed MyD88, IRAK1, IRAK2, IRAK4, TRAF6, and TAB1 expression over *β*-actin. Values are mean ± SD for at least three independent experiments performed in triplicate. ^∗∗^*p* < 0.01 versus that of the control. Compared between indicated groups, ^▲▲^*p* < 0.01.

To elucidate the underlying molecular mechanism of CPS activity, MyD88 and its downstream effectors, components of MyD88-dependent signaling pathway, were firstly accessed by western blot and/or qRT-PCR analysis. As shown in Figure 3, an evoked expression of MyD88, IRAK1, IRAK2, IRAK4, TRAF6, and TAB1 was detected following an exposure of CPS or *M. ovipneumoniae* at both of transcriptional (Figure 3(a)) and translational levels (Figures 3(b) and 3(c)) as compared with the uninfected control. Of note, a treatment of CPS antibody almost completely diminished the upregulated expression of MyD88-dependent signaling-associated proteins induced by an *M. ovipneumoniae* infection. These results imply that CPS plays a key role in *M. ovipneumoniae-*induced MyD88-dependent TLR signaling activity in sheep airway epithelial cells.

### 3.4. The CPS Triggered a MyD88-Independent TLR Signaling in Sheep Bronchial Epithelial Cells

**Figure 4:**
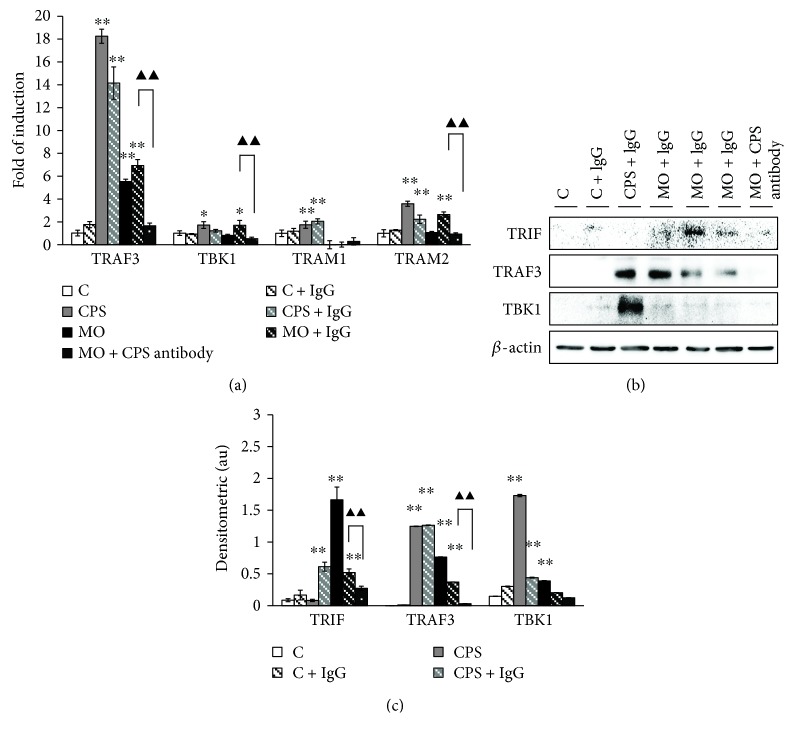
The impact of CPS on the expression of MyD88-independent TLR signaling pathway in ALI cultures of sheep bronchial epithelial cells. 4-week old ALI cultures of sheep bronchial epithelial cells were exposed to the indicated conditions for 48 h. (a) The expression of several key components of MyD88-independent signaling cascade, including the TRAF3, TBK1, and TRAM transcripts were determined by qRT-PCR assays. A significantly more abundant TRAF3 and TRAM2 mRNA was detected in ALI cultures treated with CPS/*M. ovipneumoniae*, in comparison with uninfected controls, while no evident alternations of TBK1 and TRAM1 expression were observed. (b) Expression levels of MyD88-independent signaling-associated proteins. Results showed that the expression of TRIF, TRAF3, and TBK1 were remarkably upregulated after treatments of CPS/*M. ovipneumoniae*. The immunoblots showed here were representative of three independent experiments with similar results. (c) Densitometric values are shown for TRIF, TRAF3, and TBK1 proteins over *β*-actin. Values are mean ± SD for at least three independent experiments performed in triplicate. ^∗^*p* < 0.05 and ^∗∗^*p* < 0.01 versus that of the control. Compared between indicated groups, ^▲▲^*p* < 0.01.

To further investigate whether the MyD88-independent signaling pathway was involved in CPS modulation of MO-induced proinflammatory responses in sheep epithelial cells, several key components of MyD88-independent signaling cascade, including TRAF3, TBK1, TRIF, and TRAM, were evaluated at both of transcriptional level (Figure 4(a)) and translational level (Figures 4(b) and 4(c)). An exposure to CPS or *M. ovipneumoniae* alone for 48 h resulted in a considerable elevation in the expression of TRAF3 transcript. In consistence, western blot analysis further confirmed an enhanced activation of TRAF3, TRIF, and TBK1 in the CPS- or *M. ovipneumoniae-*treated cells, despite the abundance of TBK1 transcript which was not remarkably increased. Additionally, a treatment of CPS antibody almost completely abolished the CPS- or *M. ovipneumoniae*-induced expression of above proteins, suggesting the *M. ovipneumoniae*-activated MyD88-independent signaling pathway mainly attributed by CPS in sheep bronchial epithelial cells.

### 3.5. The CPS Triggered an Activation of Nuclear Transcription Factors in Sheep Bronchial Epithelial Cells

**Figure 5:**
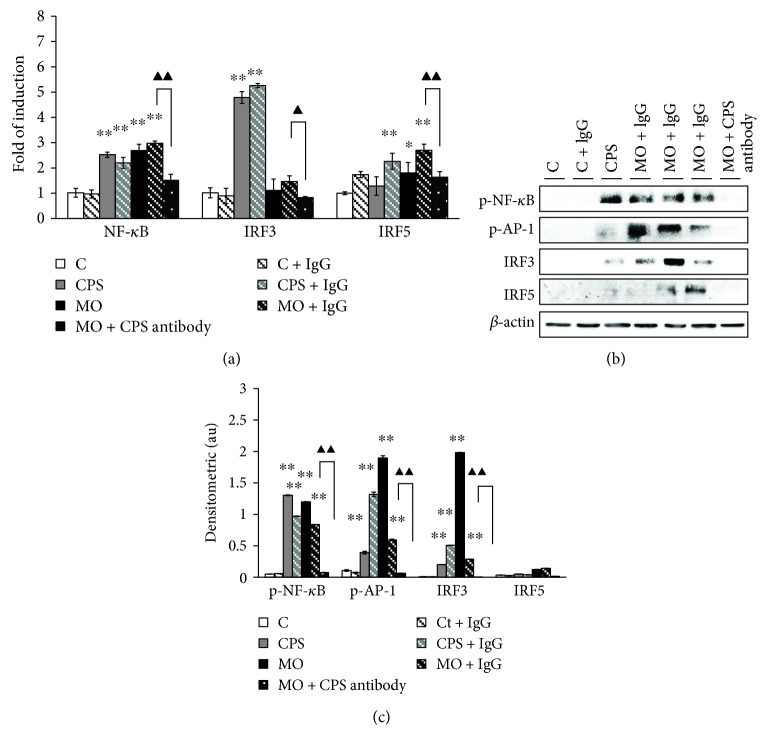
The impact of CPS on the expression of TLR signaling-associated nuclear transcription factors in ALI cultures of sheep bronchial epithelial cells. 4-week old ALI cultures of sheep bronchial epithelial cells were exposed to the indicated conditions for 48 h. (a) The expression of several key nuclear transcription factors, including the IRF5, NF-*κ*B, and IRF3 transcripts, were determined by qRT-PCR assays. An extremely more abundant IRF5 and NF-*κ*B mRNA was detected in ALI cultures treated with CPS/*MO*, while no evident alternation of IRF3 expression were observed in *M. ovipneumoniae*-infected cells. (b) Immunoblotting assay also showed the expression of p-NF-*κ*B, p-AP-1 and IRF3 protein, but not the IRF5 protein, was increased in ALI cultures upon CPS/*M. ovipneumoniae* stimulation. (c) Densitometric values are shown for p-NF-*κ*B, p-AP-1, IRF3, and IRF5 proteins over *β*-actin. These bar graphs shown here are representative of three independent experiments with similar results. ^∗^*p* < 0.05 and ^∗∗^*p* < 0.01 versus that of the control. Compared between indicated groups, ^▲^*p* < 0.05 and ^▲▲^*p* < 0.01.

The occurrence of either TLRs interacting with MyD88 or TLR4 combining to the TRIF adaptors elicits downstream signaling events. Thus, we next assessed whether TLR-associated nuclear transcription factors were inducted and activated upon CPS or *M. ovipneumoniae* stimulation. Results showed that an exposure to CPS or *M. ovipneumoniae* alone evidently upregulated the expression of IRF3, IRF5, and NF-*κ*B as determined by a RT-PCR assay (Figure 5(a)) and/or an immunoblotting assay (Figures 5(b) and 5(c)). However, in the presence of CPS antibody, the protein of IRF3, as well as the phosphorylation of NF-*κ*B and AP-1, only showed a baseline expression in response to a treatment of *M. ovipneumoniae* (Figures 5(b) and 5(c)). Of interest, no statistical difference was determined in the expression of IRF5 protein in CPS-treated cells compared with the uninfected control, despite the abundance of its transcript which was evidently increased (Figure 5).

### 3.6. The CPS Triggered the Production of Proinflammatory Mediators and Cytokines in Sheep Bronchial Epithelial Cells

**Figure 6:**
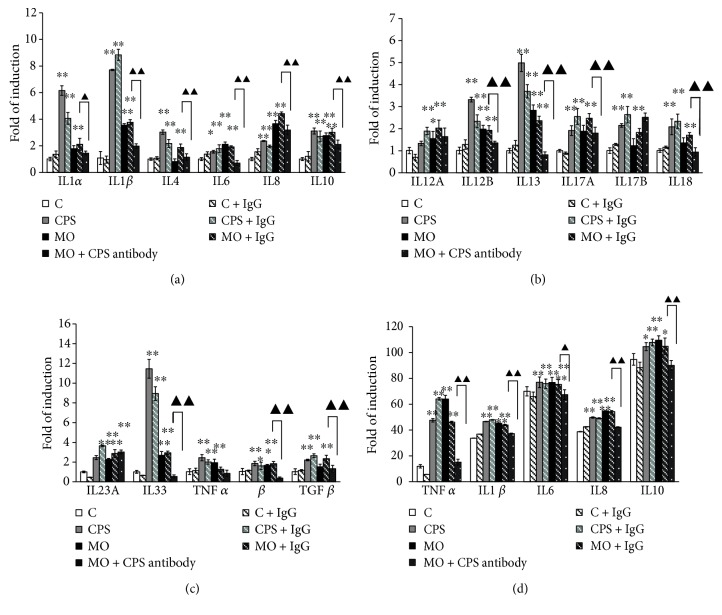
Induction of proinflammatory mediators and cytokines in the ALI cultures of sheep bronchial epithelial cells upon CPS stimulation. 4-week old ALI cultures of sheep bronchial epithelial cells were exposed to the indicated conditions for 48 h before the samples were harvested for analysis. (a–c) The mRNA expression of cytokines and chemokines including IL1*α*, IL1*β*, IL4, IL6, IL8, IL10, IL12A, IL12B, IL13, IL17A, IL17B, IL18, IL23A, IL33, TNF*α*, IFN*β*, and TGF*β* were determined by qRT-PCR assays. Compared to the control, all tested transcripts showed a significant increase after a treatment of CPS or *M. ovipneumoniae*. (d) Effect of CPS or *MO* on TNF*α*, IL1*β*, IL6, IL8, and IL10 production in the supernatant of ALI cultures were determined by ELISA. Results showed the protein expression of TNF*α*, IL1*β*, IL8, and IL10 was significantly elevated in cells upon CPS/*M. ovipneumoniae* stimulation, in comparison with uninfected controls, while no remarkable alteration of IL6 expression was observed. These bar graphs shown here are representative of three independent experiments with similar results. ^∗^*p* < 0.05 and ^∗∗^*p* < 0.01 versus that of the control. Compared between indicated groups, ^▲^*p* < 0.05 and ^▲▲^*p* < 0.01.

To further characterize the molecular mechanism of CPS activity, the concentrations of proinflammatory cytokines and chemokines were determined since they were major factors contributing to the initiation and amplification of inflammation. Relative mRNA expression data indicated that the expression of representative proinflammatory mediators including IL1*β*, IL6, IL8, IL12, and TNF*α* were remarkably elevated in cells in response to CPS or *M. ovipneumoniae* treatment (Figures 6(a)–6(c)). Intriguingly, the expression of anti-inflammatory mediators such as IL10 and TGF-*β* were also significantly induced following an exposure of CPS or *M. ovipneumoniae* alone (Figures 6(a)–6(c)). To confirm the findings from the qRT-PCR shown in Figures 6(a)–6(c), the release of TNF*α*, IL1*β*, IL6, IL8, and IL10 in culture media of the upper chamber was further quantified by an ELISA assay. Results showed that the secretion of TNF*α*, IL1*β*, IL8, and IL10 were synergistically increased upon CPS or *M. ovipneumoniae* stimulation (Figure 6(d)), therefore indicating a parallel impact between CPS and *M. ovipneumoniae* in the upregulation of proinflammatory gene expression. Additionally, CPS neutralization partially diminished cytokine accumulation and progression of *M. ovipneumoniae*-induced inflammation.

## 4. Discussion

Numerous studies have shown that inflammatory elements play a significant role in initiating and extending mycoplasma-associated diseases. However, the precise molecular mechanism following *M. ovipneumoniae* infections have not been explored and remain incompletely understood. In the present study, we identified for the first time that CPS is a key virulent component of *M. ovipneumoniae* that induces a TLR signaling-mediated inflammatory response in sheep bronchial cells, in which both CPS of *M. ovipneumoniae* and *M. pneumoniae* cells were able to trigger TLR4-MyD88-NF-*κ*B and TLR4-TRIF-IRF3 signaling activations and lead the production of varied proinflammatory cytokines, suggesting a close-knit involvement of CPS in the pathogenesis of *M. ovipneumoniae.*

Inflammation is a complex host defensive response against various harmful stimuli such as injury, radiation, and pathogens [34, 35]. The mechanism that underlies such phenomenon may be reflected in the context of activation of immune cells including monocytes and macrophages, leading to a secretion of various inflammatory mediators such as cytokines, chemokines, and subsequent nuclear signaling transcription factor [36, 37]. A baseline expression of cytokines and chemokines is supposed to be possibly involved in a sequence of immune-mediated processes, leading to the protection of host organism against pathogen invasion. However, increasing evidence has indicated an uncontrolled inflammatory response which is the main factor responsible for inflammatory diseases [37]. In most cases, polysaccharides cannot enter cells in a direct way due to the large molecular mass [38]. Emerging evidences have supported that the first step for polysaccharides to exert their functions is the recognition and identification of host cells by pattern recognition receptors (PRRs) [38, 39]. In this context, TLRs, a family of 10 identified members in sheep that are evolutionarily conserved, broadly expressed receptors which can recognize exogenous pathogen-associated molecular patterns (PAMPs) and allow the host to detect microbial infection [40].

The roles of TLRs in the initiation and activation of host immune response have been extensively investigated upon mycoplasma infection. TLR2 was the first TLR family member to be documented to response to endogenous ligands released during *Mycoplasma pneumoniae* infection, such as lipoproteins [41]. On account of the absence of a cell wall and inflammation-inducing endotoxin, lipoproteins were widely identified as an inflammation-associated factor during *M. pneumoniae* infection [42]. In general, TLR2 forms heterodimers either with TLR1 or TLR6 to attain specificity to recognize different triacylated and diacylated lipoproteins derived from *M. pneumoniae* [43]. Besides, TLR4, one major receptor for polysaccharide, plays a central role in coordinating the occurrence of innate immune response and accelerating the production of proinflammatory cytokines. Recent researches have indicated that *M. pneumonia* is capable of inducing inflammatory responses via a TLR2-independent manner, in which TLR4 and autophagy are involved [42]. Herein, we tentatively explored the expression pattern of TLRs in ALI cultures of sheep epithelial cells in response to CPS or *M. ovipneumoniae* stimulation. Consistent with above findings, significant upregulation of TLR expression including TLR1, TLR2, TLR4, and TLR6 relative to the controls was observed in sheep epithelial cells following CPS or *M. ovipneumoniae* treatment. Of particular, antibody to CPS demonstrated an ability to block the activation of TLRs, of which anti-CPS antibodies to CPS showed a potential to block the binding of *M. ovipneumoniae* to epithelial cells in vitro, therefore preventing colonization and rapid multiplication of the pathogen. These results indicated that CPS is responsible for the recognition of *M. ovipneumoniae* by epithelial cells.

It is well known that TLR signaling is initiated upon the recognition of corresponding ligand, such as polysaccharide, by its intracellular toll/interleukin-1 receptor (TIR) domain, which commonly serves as a scaffold for protein-protein interaction, resulting in a downstream signaling cascade associated with inflammation [44, 45]. MyD88 is a key adaptor protein in the activation of TLR signaling pathway, required for all identified TLRs, with an exception of TLR3. Previous studies have described that MyD88-dependent pathway played an essential role in pulmonary inflammation by stimulating various cellular and molecular events, leading to exacerbation of lung damage. Initiation of MyD88 by TLRs first triggers the recruitment of IRAK family members in sequence, including IRAK4, IRAK1, and IRAK2 [46]. IRAK is released from the receptor and subsequently leads to the activation of TNF receptor-associated factor 6 (TRAF6), which eventually regulates gene transcription of inflammatory responses by activating downstream cytosolic proteins and nuclear transcription factors such as NF-*κ*B and AP-1 [47, 48]. In agreement with this notion, our results showed that an exposure of CPS or an infection of *M. ovipneumoniae* significantly induced the upregulation of MyD88 and its downstream effectors including IRAKs, TRAF6 and TAB1, therefore indicating that a MyD88-dependent pathway might be at least in part involved in the CPS- or *M. ovipneumoniae*-induced inflammation. Besides, antibody neutralization experiments showed that the anti-CPS antibody effectively blocked *M. ovipneumoniae*-induced activation of MyD88-dependent signaling pathway from ALI cells, therefore suggesting that the CPS of this pathogen may be the main component responsible for inflammatory responses induced by *M. ovipneumoniae* infections.

Alternatively, it has been described that TLR stimulation can activate a MyD88-independent alternative pathway mediated by TIR-domain-containing adapter-inducing interferon-*β* (TRIF) and IRF3. TRIF, one of the TIR domain-containing adaptors, is a MyD88 homologous protein, which is specifically implicated in the TLR3- and TLR4-mediated MyD88-independent pathway [41]. Mechanistically, upon the activation, an array of downstream protein including TRAF3 and TBK1 is introduced. As the key transcription factor, IRF3 is then phosphorylated by TBK1 and is transferred into nucleus and bind with the *ifn* promoter, triggering *ifn* transcription [49]. Our results showed that the occurrence of inflammation induced by both CPS and *M.ovipneumoniae* was linked to a MyD88-independent signaling pathway due to the fact that TRIF and its associated downstream proteins were simultaneously upregulated in sheep epithelial cells following CPS and *M. ovipneumoniae* treatment. Meanwhile, the expression of *M. ovipneumoniae*-induced TRIF, TRAF3, TBK1, and TRAM expression was found to be markedly attenuated in the presence of CPS antibody, therefore indicating an important role of CPS in immune responses of *M. ovipneumoniae* during infections.

Given the fact of both MyD88-dependent and MyD88-independent pathways were involved in the CPS of *M. ovipneumoniae* induced inflammation in ALI cultures of sheep bronchial epithelial cells, the underlying nuclear mechanisms by which CPS triggered inflammation were thus further explored. In the downstream of above signaling pathways, phosphorylation is pivotal for mediating nucleus translocation and functional transcription. Nuclear factor-*κ*B (NF-*κ*B) and activator protein-1 (AP-1) are two important downstream mediators of immediate early gene expression which couples extracellular signals to gene-activating events associated with a variety of inflammatory, apoptotic, and immune responses [50]. NF-*κ*B is normally comprised of a 50 kDa subunit (p50) and a 65 kDa subunit (p65) and is located within the cytoplasm in an inactive form via binding a member of the inhibitory kappa B (I*κ*B) family [51]. A characteristic feature of NF-*κ*B is its phosphorylation and translocation by posttranslational mechanisms related to the dissociation of I*κ*B complex [52, 53]. In addition, AP-1 is a ubiquitous transcription factor that is mainly composed of the *jun* and *fos* gene products which form diverse homo (*Jun/Jun*) or heterodimeric (*Jun/Fos*) complex [54]. Once activated, the dimers are phosphorylated and translocated into the nucleus to bind target genes and subsequently initiate specific gene transcription [55]. As expected, our data showed that the activity of NF-*κ*B and AP-1 was significantly increased in CPS or *M. ovipneumoniae* treated cells, which was partially inhibited by the treatment of CPS antibody. On the other hand, the activity of IRF3, a downstream nuclear transcription factor mediated by TRIF-dependent pathway, was also detected. In line with this hypothesis, an exposure of CPS or *M. ovipneumoniae* induces the increased expression of IRF3, thus initiating an immediate late gene transcription. To confirm the role of CPS in *M. ovipneumoniae*-activated signaling events, above key proteins were further analyzed in the presence of CPS antibody. As expected, the presence of anti-CPS antibodies led notably to the reduction of *M. ovipneumoniae*-induced phosphorylation of NF-*κ*B and AP-1, as well as the suppression of the nuclear translocations of IRF3 and IRF5. Taken as a whole, these results strongly suggest that both MyD88-dependent and MyD88-independent signaling pathways play key roles in the response of sheep airway epithelial cells upon CPS or *M. ovipneumoniae* infection, especially CPS plays a key role in *M. ovipneumoniae*-activated TLR signaling in this cell type. However, further studies are needed to elucidate the relationship between MyD88-dependent and MyD88-independent pathways in order to uncover the precise mechanism underlying complex interactions in innate immune signaling.

Upon a stimulation of *M. ovipneumoniae*, inflammatory cytokines activate the immune system in response to “danger” and increase the efficiency of an immune response; the underlying mechanisms of immunomodulators are thought to occur via the activation of epithelial cells and macrophages, which secret an array of immune-associated factors, including TNF*α*, IL1*β*, IL6, IL8, and IL10, which are important in mediating both lung defense and inflammation [56]. Respectively, TNF*α* is implicated in acute phase of inflammation by activating epithelial cells and subsequent conducing to the generation of other inflammatory mediators [57]. As an important modular in the upper reaches of the immune response, IL1*β* participates in epithelial repair and amplifies the inflammatory cascade [16]. IL8 possesses neutrophil chemotactic and activating activities and is highly related to acute lung inflammatory injury. As a consequence, the study of cytokine and chemokine profiles may reveal the characteristics of innate immune mechanisms in epithelial cells following a mycoplasma infection. Indeed, a previous study has demonstrated that *M. pneumoniae* infection induces proinflammatory cytokine expression in human lung carcinoma cell line (A549) including IL1*β*, IL6, IL8, and TNF*α* [58]. In addition, using human monocyte cell line (THP-1), Shimizu et al. have revealed a that wild-type stain of *M. pneumoniae* with cytoadherence ability is capable of inducing quite a number of proinflammatory cytokines such as TNF*α* and IL1*β* [59]. These data suggest that the proinflammation ability is involved in the pathogenesis of *M. pneumoniae* infection. Consistent with above conclusions, our results showed that the introduction of CPS or *M. ovipneumoniae* led to the upregulation of various inflammatory mediators, particularly including IL1*β*, TNF*α*, and IL8, which are widely involved in the regulation of inflammation. To further identify the underlying immune mechanisms of CPS during *M. ovipneumoniae*-induced inflammation, a CPS antibody was used to continuously neutralize *M. ovipneumoniae* and assess the production of proinflammatory cytokines. Results showed that neutralization of CPS alleviated the *M. ovipneumoniae*-induced production of inflammatory mediators and effectors, therefore suggesting the close-knit involvement of CPS in *M. ovipneumoniae*-induced inflammation.

However, an excessive secretion of proinflammatory cytokines by immoderating immune response activation could subsequently trigger systemic inflammatory response and eventually contributing to the generation of amplification loops in the inflammatory cascade [60, 61]. At present, imbalance between proinflammation and anti-inflammation is well known to play an essential role in the pathogenic process of pneumonia. It has been reported previously that IL1*β* is responsible for its own stimulation, thus resulting in an activation and amplification of IL-1R-mediated initiation of MyD88-dependent signaling pathways, eventually leading to the release of more proinflammatory cytokines including IL1*β* itself and hence an escalated host inflammatory response [13]. Besides, some other oxidants and inflammatory mediators such as TNF*α* are recorded to play a critical role in the inflammatory cascades as well [62], in which the dysregulation of TNF*α* is widely associated with the pathogenesis of several diseases, including septic shock, rheumatoid arthritis, and atherosclerosis [28, 63]. Therefore, the production of proinflammatory cytokines, such as IL1*β* and TNF*α*, is required to be strictly regulated in maintaining the balance between the proinflammatory and anti-inflammatory responses. As anticipated, our results showed that the mRNA expression of IL10 and TGF*β*, two typical anti-inflammatory cytokines, were considerably enhanced following CPS or *M. ovipneumoniae* treatment. Noteworthy, the CPS-neutralized group showed lower levels of IL10 and TGF*β* expression compared with the *M. ovipneumoniae*-treated group, therefore indicating CPS of *M. ovipneumoniae* could modulate the host immune function via releasing a mixture of both pro- and anti-inflammatory cytokines and chemokines from sheep epithelial cells.

**Figure 7:**
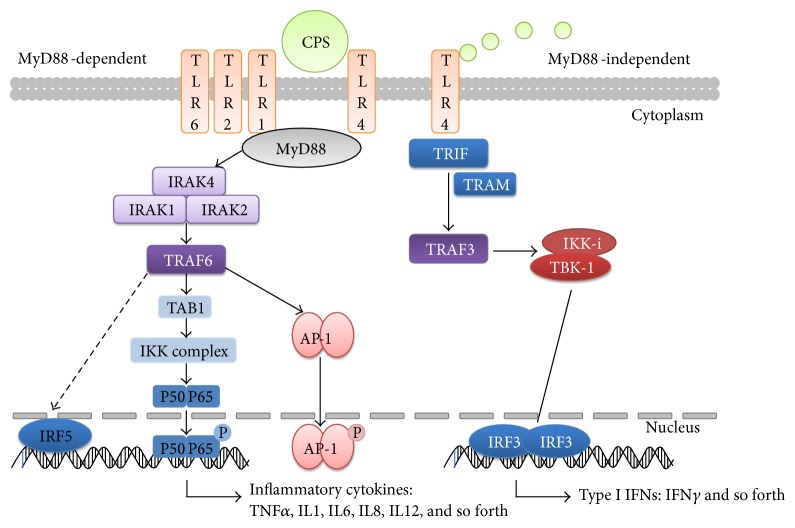
Scheme shows a possible mechanism of CPS-induced proinflammatory responses in ALI cultures of sheep bronchial epithelial cells. Both MyD88-dependent and MyD88-independent TLRs signaling pathways may play key roles in the sheep airway epithelial cells upon CPS or *M. ovipneumoniae* treatment.

Collectively, results from our study using an ALI culture of primary sheep bronchial epithelial cells suggest that CPS, which is the major capsular component of *M. ovipneumoniae*, might serve as main endogenous PAMPs of this pathogen to activate the epithelial inflammation. In this respect, CPS of *M. ovipneumoniae* could trigger inflammatory responses in sheep bronchial epithelial cells through a mechanism by which it activated both MyD88-dependent and MyD88-independent signaling pathways and subsequently enhanced the production of various pro-inflammatory cytokines, such as IL1*β* and TNF*α*, and meanwhile increased the expression of anti-inflammatory cytokines, such as IL10 and TGF*β* (Figure 7()). This attribution of CPS makes it a promising vaccine candidate for *M. ovipneumoniae* infection as proinflammation may be an important step in the establishment of disease. Besides, considering inhibition of inflammatory mediators serves as a key strategy to control inflammation; specific agents that can suppress the transcription and translation of inflammation-associated genes may have a therapeutic potential for *M. ovipneumoniae* infections.

## Figures and Tables

**Table 1:** Sheep gene specific primers for qRT-PCR.

Gene	Forward (primer sequence 5′-3′)	Forward (primer sequence 5′-3′)
*β*-actin	GCTCTCTTCCAGCCTTCCTT	AGGTCTTTGCGGATGTCG
TLR1	CAGTGGGATTCAGTAGGT	TTGTTTCCAGGGATAAGT
TLR2	TCCTGTTGCTCCTGCTCAC	CTTCCTGGGCTTCCTCTTG
TLR4	TTTGTCCCTGAACCCTTTAGAA	AAACCAGCCAGACCTTGAATAC
TLR6	CAGTGAATAGTTTAGGGTGCTTACA	CGTTGGTCTTCCAGTGAGTC
MyD88	ATGGTGGTGGTTGTCTCTGAC	GGAACTCTTTCTTCATTGGCTTGT
IRAK1	TGGACACCGACACCTTCAG	CTGCCTCCTCCTCAACCAAG
IRAK2	TATGGCTGGATGAAGAACCA	CAGGGAGGGAGAATGAATGA
IRAK4	TGTGCTACCTGACTCCTCAAG	ACTCCAAACCCTCCTTCTCC
TRAF6	TCAGAGAACAGATGCCCAAT	GCGTGCCAAGTGATTCCT
TAB1	GGGTCTTCAATGGCTACGAT	TCCAACGCCTTGAGTCTGT
TRAF3	CCCTGGAGAAGAAGGTTTCC	CGATGACTCGCTGTAAATGG
TBK1	TGTATCTCCGACTGCCTGCT	GGTTCTGCTCTCCACTCACAG
TRAM1	CTGCCACAGAAGAACAAG	AAGCCCTCCACAAGATAG
TRAM2	CGTTAGTGTCCCTTATCGTG	CGTCAGTTCATCAGCACCT
IRF5	GCACCCTATTCTTTACCCAA	TCAGGCAGGAGTTGTTCG
NF-*κ*B	CGAGGATGATGAGAATGG	CAGGAACACGGTTACAGG
IRF3	GAAGGAAGTGTTGCGTTTAGC	TGTCTGCCATTGTCTTGAGC
IL1*α*	CCCTGGATACCTCGGAAAC	AAACTCAACCGTCTCTTCTTCA
IL1*β*	TCTCCCTAAAGAAGCCATAC	AGCGTCTCAGCACGAATA
IL4	CGTAAGAAGGGCGAGCAG	AGGTGGAGGTAGGGTCAGC
IL6	TTGAGGGAAATCAGGAAACTGT	TGTGGCTGGAGTGGTTATTAGA
IL8	GCTGGCTGTTGCTCTCTTG	GTGGAAAGGTGTGGAATGTGTT
IL10	TTTCCCTGACTGCCCTCTAAT	CTCCCTGGTTTCTCTTC
IL12A	GGGCATTGTCTGTCTTCT	TCTTCCAGGGAGGGTTTC
IL12B	ATTCTCGGCAGGTGGAAGT	CTGAGGTTTGGTCTGTGAAGAG
IL13	CCTATGCGTCTGCTTCTC	ATCCTCTTGGTCCTGTGG
IL17A	AGCACAAGCCCATCCATC	CCTGCCTTCACAAGAGCC
IL17B	TGGTCGGCAGACTAAGAT	CAAGAATACTGGAGTGGGTT
IL18	AAATGGCGAAGACCTGGAATC	TCCCTGGCTAATGAAGAGAACTT
IL23A	AACCAGACGCACAGAACA	AGCAGCAGCATCACAACT
IL33	AGGGCTTCACCTTGGGTAATA	CACCTTGTCTTTCTCTTGGTCTC
TNF*α*	CCCAACTCCCTCTGTTTATGTT	GGACACCTTGACCTCCTGAATA
IFN*β*	AGAACCTCCTGTGGCAGTTAC	CAATACGGCATCTTCCTTCC
TGF*β*	GTTCTTCAACACGTCCGAG	CACGTGCTGCTCCACTTTTA
